# Supplementary material for: The Multifaceted Gene 275 Embedded in the PKS-PTS Gene Cluster Was Involved in the Regulation of Arthrobotrisin Biosynthesis, TCA Cycle, and Septa Formation in Nematode-Trapping Fungus Arthrobotrys oligospora
Source: J Fungi (Basel). 2022 Nov 29;8(12):1261. doi: 10.3390/jof8121261 (PMC9780802; doi:10.3390/jof8121261)
Supplement: Supplementary file 1 [file jof-08-01261-s001.zip › JOF-275-Supporting information.pdf]

Supporting Information

The Multifaceted Gene *275* Embedded in the PKS-PTS Gene Cluster was Involved in the Regulation of Nematicidal Activity, Arthrobotrisin Biosynthesis, TCA Cycle, and Septa Formation of Nematode-Trapping Fungus *Arthrobotry oligospora*

Jiao Zhou[1,2], Qun-Fu Wu,[1,2] Shu-Hong Li,[1] Jun-Xian Yan,[1] Li Wu,[1] Qian-Yi Cheng,[1] Zhi-Qiang He, [1] Xu-Tong Yue,[1] Ke-Qin Zhang,[1] Long-Long Zhang,[1] Xue-Mei Niu,[1]*

State Key Laboratory for Conservation and Utilization of Bio-Resources & Key Laboratory for Microbial Resources of the Ministry of Education, School of life Sciences, Yunnan University, Kunming 650091, People's Republic of China

Jiao Zhou, Qun-Fu Wu, contributed equally to this work.

Table S1. List of primers for *275*

Gene	Primer	Sequence (5′-3′)
275	275up-F	GAGCTCGGTACCAAGGCCCGGGACTTATCAATTAGGGCTCAC
	275up-R	AGGCCTGATCATCGATGGGCCCAACAAATCGGAATAGGGT
	275down-F	TCTAGAGGATCCCCCGACTAGTCCCAGCATATTAGATAGAG
	275down-R	CACGAAGCTTGCATGCCTGCAGGTTCTCACATAATCCGTCA
	275yz-5f	CGTGAATTCAGCCTCATGAGC
	275yz-3r	AGGTACCACGTTGACGAAGG

Table S2. List of primers for OE-*275*

Gene	Primer	Sequence (5′-3′)
275	275-5f	ATGTTCACTTCTCTTCGCTTTCTATG
	275-3r	TTATAGAATAACACAATCACTTCCAAGTTTGGATAGAG
	PUC-5f	GAAGAGAAGTGAACATATCGATGCTTGGGTAGAATAGGT
	PUC-3r	TGTGTTATTCTATAACCACTTAACGTTACTGAAATCATC
	275yz-5f	AAGCAAGGTAAGTGAACGACCC
	275yz-3r	AACTCCGGAGCTGACATCG
	275RT-5f	CTCTAAGTTCAAGCATCGGAC
	275RT-3r	ACGGATAGAGGAGGCAATG
	β-tubulin-5f	CCACCTTCGTCGGTAACTC
	β-tubulin-3r	TCGTCCATACCCTCACCAG
